# Effects of vitamin D receptor polymorphisms on urolithiasis risk: a meta-analysis

**DOI:** 10.1186/1471-2350-14-104

**Published:** 2013-10-06

**Authors:** Pan Zhang, Wei Nie, Hong Jiang

**Affiliations:** 1 Department of Nephrology, the First People’s Hospital of Jingzhou City, the First Hospital of Yangtze University, Jingzhou, Hubei Province 434000, China; 2 Department of Respiratory Disease, Shanghai Changzheng Hospital, Second Military Medical University, Shanghai 200003, China

**Keywords:** Urolithiasis, Vitamin D receptor, Polymorphism, Meta-analysis

## Abstract

**Background:**

Several studies analyzed the associations of *Vitamin D receptor* (*VDR*) polymorphisms with urolithiasis risk in different ethnic groups. However, the results were inconclusive. To evaluate a more precise estimation of the relationship, a meta-analysis was performed.

**Methods:**

Pubmed, EMBASE, Wanfang Database, China National Knowledge Infrastructure (CNKI) and Weipu Database were searched. Data were extracted independently by two investigators. Odds ratios (ORs) with 95% confidence intervals (CIs) were used to assess the strength of associations.

**Results:**

Twenty-three case–control studies were included in this meta-analysis. Significant associations between *ApaI*, *BsmI*, *FokI*, and *TaqI* polymorphisms and urolithiasis risk were observed. However, sensitivity analyses for *BsmI* and *FokI* polymorphisms indicated that the results were not reliable and credible. In addition, there was a significant association of the *ApaI*-*TaqI* haplotype with urolithiasis risk.

**Conclusions:**

This meta-analysis suggested that *ApaI* and *TaqI* polymorphisms in *VDR* gene were associated with urolithiasis risk.

## Background

Urolithiasis is one of the most prevalent uronephrologic disorders and affects approximately 10% of individuals in western countries [1]. The incidence of urolithiasis is increasing. For example, in the US the prevalence has risen from 3.2% to 5.2% in just over two decades from the mid-1970s to the mid-1990s [2]. Previous studies evidenced the importance of genes in this disease. Studies of kidney stone-forming twins demonstrated a higher concordance for kidney stones in monozygotic than in dizygotic twins [3]. Additionally, a family history was reported to increase the disease risk (2.57 times higher) in males [4]. Thus, it is important to identify the gene variants contributing to urolithiasis pathogenesis.

Recently, Elkoushy and coworkers found that patients with urolithiasis had a high prevalence of inadequate vitamin D [5]. Expression and nuclear activation of the Vitamin D receptor (VDR) are necessary for the effects of vitamin D. Therefore, VDR was implicated in urolithiasis. In genetic hypercalciuric stone-forming (GHS) rats, Yao et al. [6] found that VDR mRNA levels were higher in kidney compared with wild-type controls. In addition, Favus et al. [7] showed that the level of VDR in peripheral blood monocytes was twofold greater in male calcium oxalate stone formers than in controls. Taken together, these results suggested that VDR may play an important role in the pathogenesis of urolithiasis.

The human *VDR* gene is located on chromosome 12q12-14. Four single nucleotide polymorphisms (SNPs) of the *VDR* gene have been widely studied [8]. *ApaI*, *BsmI*, and *TaqI* are located between the 8 and 9 exons in the 3′-untranslated region (UTR), and shown to be in strong linkage disequilibrium (LD) [9]. Another SNP is the *FokI*, which is located at the translation starting codon. Many studies investigated the associations between these polymorphisms of *VDR* gene and the risk of urolithiasis [10-32]. However, the results were inconclusive. The inconsistent results were possibly due to the low statistical powers of individual studies. The method of meta-analysis could provide a quantitative approach for combining the results of various studies with the same topic. Therefore, we performed this meta-analysis to address the precise relationship between the *VDR* gene variants and urolithiasis risk.

## Methods

### Publication search

We performed a systematic search of Pubmed, EMBASE, Wanfang Database, China National Knowledge Infrastructure (CNKI) and Weipu Database to find relevant studies. The search terms were used as follows: (urolithiasis or kidney stone or kidney stone disease) and (Vitamin D receptor or VDR) and (polymorphism or mutation or variant). Last search was updated in October, 2012. No language restriction was imposed. The reference lists of searched articles and relevant reviews were all perused to find additional eligible studies.

### Inclusion and exclusion criteria

Two reviewers (Zhang and Nie) independently screened titles and abstracts of all studies for relevancy. Disagreements were resolved by discussion. Studies included in this meta-analysis based on the following selection criteria: (1) evaluation of the *ApaI*, *BsmI*, *TaqI*, and *FokI* polymorphisms in *VDR* gene and urolithiasis risk, (2) using a case–control design, and (3) genotype distributions in both cases and controls should be available for estimating an odds ratio (OR) and 95% confidence interval (CI).

Studies were excluded if one of the following existed: (1) the *VDR* polymorphisms were not analyzed or the outcome was not urolithiasis risk, (2) not case–control studies, such as the design based on family or sibling pairs, (3) not reported genotype frequencies or number, (4) abstracts or reviews, and (5) non-clinical study. For overlapping studies, the one with the largest sample size was included.

### Data extraction

The full manuscripts of eligible studies were reviewed by two investigators (Zhang and Nie) independently. Any discrepancy was resolved by discussion or a third author (Jiang) would assess the articles. The following information was collected from each study: the first author’s name, year of publication, original country, ethnicity, age group, hypercalciuria in the urolithiasis group, composition of stone, sample size, the polymorphisms in *VDR* gene, genotyping method, and genotype number in cases and controls. We contacted the corresponding authors if more data was needed.

### Quality assessment

The quality of the studies was assessed by two investigators (Zhang and Nie) independently. The predetermined criteria were modified from a previous review [33]. These scores were based on traditional epidemiological considerations and genetic issues. Scores ranged from zero (lowest) to ten (highest). Disagreement was settled by discussion. Articles scoring < 5 were defined as low quality, and those ≥ 5 were defined as high quality.

### Statistical analysis

When the data from at least three similar studies were available, meta-analysis was performed. The strength of the associations between the *ApaI*, *BsmI*, *FokI*, and *TaqI* polymorphisms and urolithiasis risk was measured by ORs and 95% CIs. OR1, OR2, and OR3 were calculated for the genotypes: 1) AA vs. aa (OR1), aA vs. aa (OR2), and AA vs. aA (OR3) for the *ApaI*, 2) bb vs. BB (OR1), bB vs. BB (OR2), and bb vs. bB (OR3) for the *BsmI*, 3) ff vs. FF (OR1), fF vs. FF (OR2), and ff vs. fF (OR3) for the *FokI*, and 4) tt vs. TT (OR1), tT vs. TT (OR2), and tt vs. tT (OR3) for the *TaqI*, respectively. The statistical significance of OR was analyzed by *Z* test. These pairwise differences were used to indicate the most appropriate genetic model as follows: if OR1 = OR3 ≠ 1 and OR2 = 1, then a recessive model was suggested; if OR1 = OR2 ≠ 1 and OR3 = 1, then a dominant model was suggested; if OR2 = 1/OR3 ≠ 1 and OR1 = 1, then a complete overdominant model was suggested; if OR1 > OR2 > 1 and OR1 > OR3 > 1 (or OR1 < OR2 < 1 and OR1 < OR3 < 1), then a codominant model was suggested [34,35]. Once the best genetic model was identified, this model was used to collapse the three genotypes into two groups (except in the case of a codominant model) and to pool the results again. The pooled OR estimate of each study was calculated by the random-effects model.

The between-study heterogeneity was assessed by the Chi square-test based Cochrane *Q*-test and *I*^2^ test. *I*^2^ values of 25%, 50%, and 75% were nominally assigned as low, moderate, and high estimates. Departure from Hardy-Weinberg equilibrium (HWE) in controls was tested by the Chi-square test. Subgroup analyses were conducted by ethnicity, calciuria level, and age group. Sensitivity analyses were performed by excluding the studies not in HWE and the studies with low quality, respectively. Funnel plot was used to assess potential publication bias. Publication bias was also investigated statistically via Egger’s test [36].

All statistical tests were performed by using STATA 11.0 software (Stata Corporation, College Station, TX). A *P* value < 0.05 was considered statistically significant, except for test of heterogeneity where a level of 0.10 was used.

## Results

### Study characteristics

**Figure 1:**
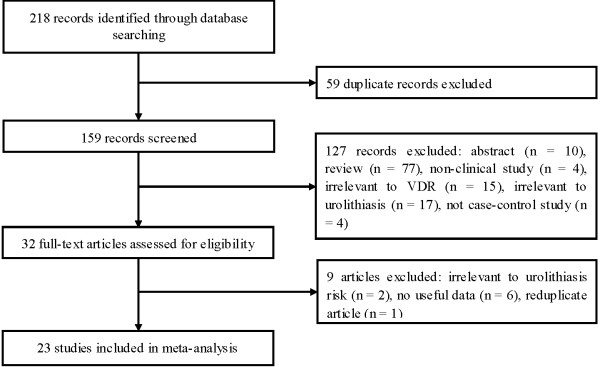
Flow of study identification, inclusion, and exclusion.

Figure 1 outlines our study selection process. A total of 218 articles were identified after an initial search. Fifty-nine duplications were excluded. After reading the titles and abstracts, 127 articles were removed owing to abstracts, reviews, non-clinical studies, not case–control studies, and irrelevant to urolithiasis or *VDR* polymorphisms. After reading the full texts of the remaining 32 articles, 9 articles were excluded due to irrelevant to urolithiasis risk, no useful data, and reduplicate study. Finally, a total of 23 case–control studies met our inclusion criteria. There were 11 studies on *ApaI*, 8 on *BsmI*, 14 on *FokI*, 13 studies on *TaqI*. Four studies reported the haplotype of *ApaI* and *TaqI* polymorphisms. There were 14 studies of Asians and 9 studies of Caucasians. Seventeen studies were performed in adults, 3 in children, and 1 did not offer detailed information. Three studies only included patients with hypercalciuria, 6 studies included hypercalciuria patients partly and the data for these patients could be extracted, and 14 studies did not report detailed information. Quality scores for the each study ranged from 3 to 6. The characteristics of each study are presented in Table 1.

**Table 1:** Characteristics of the case–control studies included in meta-analysis

				**Age group**	**Hypercalciuria in case group**		**Case (n)**	**Control (n)**	***VDR *polymorphisms**	**Genotyping method**	**Quality score**
**First author**	**Year**	**Country**	**Ethnicity**			**Composition**			**(HWE)**		
Ruggiero [10]	1999	Italy	Caucasian	Adult	Mixed*	NA	27	150	*Bsm*I (No)	PCR-RFLP	4
Jackman [11]	1999	USA	Caucasian	NA	All	Calcium	17	37	*Taq*I (No)	PCR-RFLP	3
Chen a [12]	2001	China	Asian	Adult	NA	Calcium	124	90	*Bsm*I (No)	PCR-RFLP	4
Chen b [13]	2001	China	Asian	Adult	NA	Calcium	146	90	*Fo*kI (Yes)	PCR-RFLP	4
Nishijima [14]	2002	Japan	Asian	Adult	NA	Calcium	83	83	*Apa*I (Yes), *Taq*I (Yes)	PCR-RFLP	5
Mossetti [15]	2003	Italy	Caucasian	Adult	NA	Calcium	220	114	*Bsm*I (Yes), *Taq*I (No)	PCR-RFLP	5
Ozkaya [16]	2003	Turkey	Caucasian	Children	All	Calcium	64	90	*Apa*I (No), *Bsm*I (Yes), *Taq*I (Yes)	PCR-RFLP	4
Wang a [17]	2003	China	Asian	Adult	Mixed*	Calcium	150	80	*Apa*I (Yes), *Fo*kI (Yes), *Taq*I (Yes)	PCR-RFLP	3
Relan [18]	2004	India	Asian	Adult	Mixed*	Calcium	150	100	*Bsm*I (No), *Fo*kI (No)	PCR-RFLP	3
Rendina [19]	2004	Italy	Caucasian	Adult	All	Calcium	159	124	*Apa*I (Yes), *Fo*kI (Yes)	PCR-RFLP	6
Hu [20]	2004	China	Asian	Adult	Mixed*	Calcium	186	90	*Apa*I (Yes), *Fo*kI (Yes), *Taq*I (Yes)	PCR-RFLP	5
Bid a [21]	2005	India	Asian	Adult	Mixed*	Calcium	138	166	*Fo*kI (No)	PCR-RFLP	4
Bid b [22]	2005	India	Asian	Children	NA	Calcium	50	60	*Fo*kI (Yes)	PCR-RFLP	5
Gunes [23]	2006	Turkey	Caucasian	Adult	NA	Calcium	110	150	*Apa*I (Yes), *Bsm*I (Yes), *Taq*I (Yes)	PCR-RFLP	6
Liu [24]	2007	China	Asian	Adult	NA	Calcium	235	231	*Fo*kI (Yes)	PCR-RFLP	6
Moyano [25]	2007	Spain	Caucasian	Adult	NA	Calcium	51	21	*Apa*I (Yes), *Bsm*I (Yes), *Taq*I (Yes)	PCR-RFLP	3
Seyhan [26]	2007	Turkey	Caucasian	Children	Mixed*	Calcium	80	40	*Taq*I (No)	PCR-RFLP	4
Wang b [27]	2009	China	Asian	Miexd	NA	Calcium	90	90	*Apa*I (No), *Fo*kI (No)	PCR-RFLP	5
Mittal [28]	2010	India	Asian	Adult	NA	NA	125	150	*Apa*I (Yes), *Fo*kI (No), *Taq*I (No)	PCR-RFLP	5
Seo [29]	2010	Korea	Asian	Miexd	NA	Calcium	102	535	*Apa*I (No), *Fo*kI (No), *Taq*I (No)	PCR-RFLP	5
Basiri [30]	2012	Iran	Caucasian	Adult	NA	Calcium	106	109	*Fo*kI (No), *Taq*I (No)	PCR-SSCP	5
Wang c [31]	2012	China	Asian	Adult	NA	Calcium	464	450	*Apa*I (Yes), *Bsm*I (Yes), *Fo*kI (Yes)*, Taq*I (Yes)	PCR-RFLP	6
Ruan [32]	2012	China	Asian	Adult	NA	Calcium	169	156	*Fo*kI (No)	PCR-RFLP	4

*Data for hypercalciuria patients could be extracted.

HWE, Hardy-Weinberg equilibrium; PCR, polymerase chain reaction; RFLP, restriction fragment length polymorphism; SSCP, single-strand conformational polymorphism; NA, not available.

### Quantitative data synthesis

#### ***VDR ApaI polymorphism***

**Figure 2:**
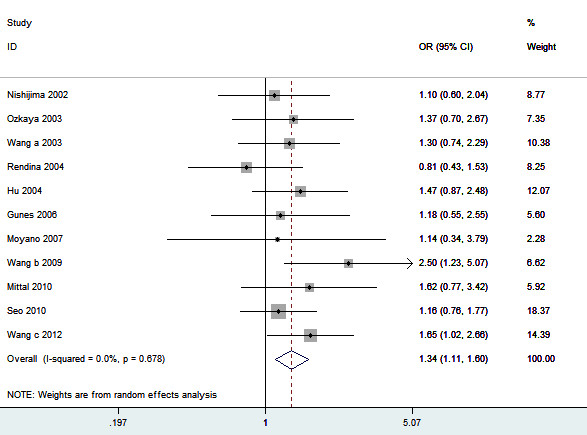
**Meta-analysis for the association between urolithiasis risk and the *VDR ApaI *polymorphism.**

**Figure 3:**
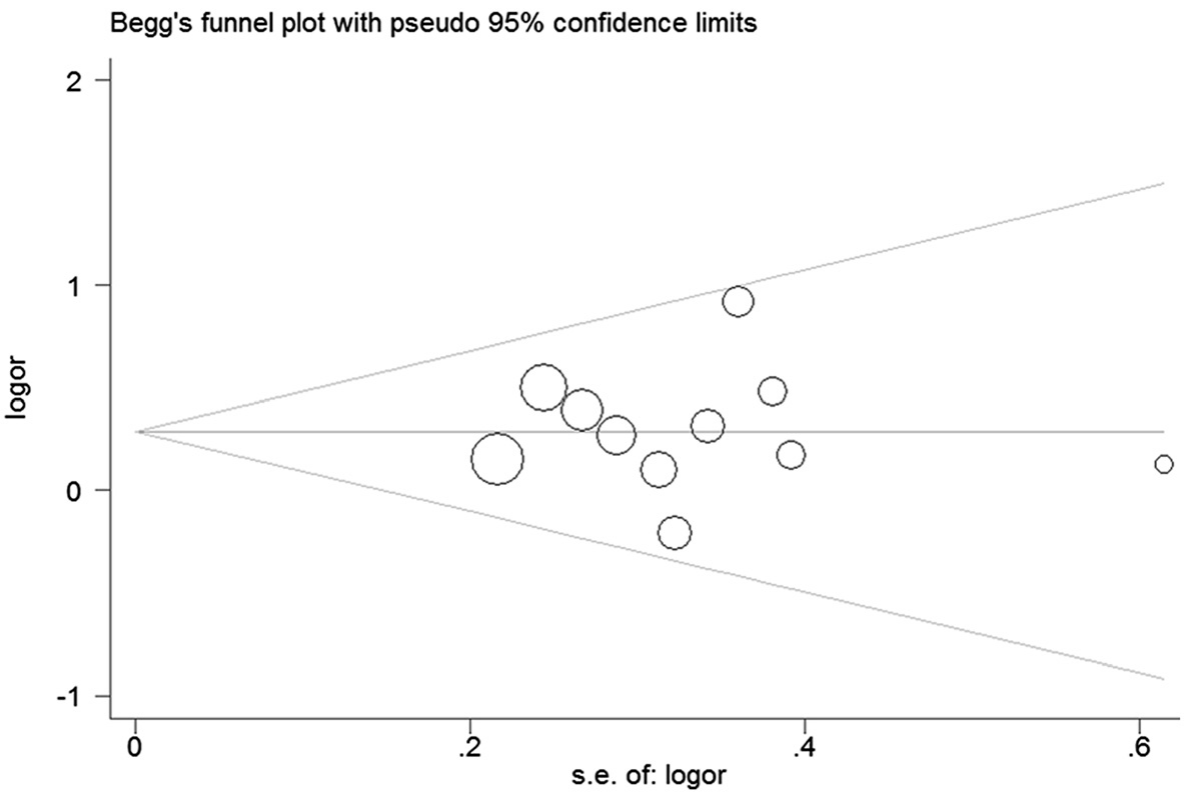
**Funnel plot for publication bias in selection of studies on the *VDR ApaI *polymorphism.**

Eleven studies determined the association between *ApaI* polymorphism and urolithiasis risk. Total sample sizes in urolithiasis and control groups were 1584 and 1853. The estimated OR1, OR2 and OR3 were 1.47, 1.30, and 1.04, respectively (Table 2). These estimates suggested a dominant genetic model, and therefore AA and aA were combined and compared with aa. As shown in Figure 2, the pooled OR was 1.34 (95% CI 1.11 – 1.60, *P* = 0.002). In the subgroup analysis by ethnicity, a significant association was found among Asians (OR = 1.43, 95% 1.16 – 1.75, *P* < 0.001) but not among Caucasians (OR = 1.08, 95% 0.74 – 1.57, *P* = 0.69). Subgroup analysis was also performed according to the calciuria level. However, no significant increased risk of urolithiasis was found among hypercalciuric patients (OR = 1.24, 95% CI 0.86 – 1.81, *P* = 0.25) (Table 2). In the subgroup analysis by age group, a significant association was observed among adults (OR = 1.30, 95% 1.04 – 1.62, *P* = 0.02). Sensitivity analysis was performed by excluding the studies that did not show HWE. The result was statistically significant (OR = 1.30, 95% CI 1.04 – 1.62, *P* = 0.02). Sensitivity analysis was also performed by excluding the low quality studies. The result was similar (OR = 1.34, 95% CI 1.09 – 1.66, *P* = 0.006). The shape of the funnel plot showed symmetric (Figure 3). Egger’s test did not indicate significant publication bias (*P* = 0.914).

**Table 2:** Summary of different comparative results

		**Sample size**		**No. of studies**	**Test of association**				**Heterogeneity**		
**Polymorphisms**	**Study**	**Case**	**Control**		**OR (95% CI)**	***Z***	***P *Value**	**Model**	***χ***^**2**^	***P *Value**	***I***^**2 **^**(%)**
*Apa*I											
AA vs. aa	Overall	846	1048	11	1.47 (1.11-1.94)	2.66	0.008	R	13.03	0.220	23.0
aA vs. aa	Overall	1059	1368	11	1.30 (1.06-1.60)	2.54	0.010	R	10.98	0.360	9.0
AA vs. aA	Overall	1263	1290	11	1.04 (0.76-1.43)	0.25	0.810	R	26.31	0.003	62.0
AA + aA vs. aa	Overall	1584	1853	11	1.34 (1.11-1.60)	3.12	0.002	R	7.49	0.680	0.0
AA + aA vs. aa	Asian	1200	1468	7	1.43 (1.16-1.75)	4.57	<0.001	R	4.57	0.600	0.0
AA + aA vs. aa	Caucasian	384	385	4	1.08 (0.74-1.57)	0.40	0.690	R	1.32	0.730	0.0
AA + aA vs. aa	Hypercalciuria	295	262	4	1.24 (0.86-1.81)	1.15	0.250	R	3.10	0.380	3.0
AA + aA vs. aa	Adult	1328	1148	8	1.30 (1.04-1.62)	2.33	0.020	R	3.99	0.780	0.0
AA + aA vs. aa	HWE	1328	1148	8	1.30 (1.04-1.62)	2.33	0.020	R	3.99	0.780	0.0
AA + aA vs. aa	High quality	1319	1662	8	1.34 (1.09-1.66)	2.75	0.006	R	7.41	0.390	6.0
*Bsm*I											
bb vs. BB	Overall	841	855	8	1.79 (0.98-3.26)	1.89	0.060	R	18.93	0.008	63.0
bB vs. BB	Overall	611	609	8	1.85 (1.03-3.32)	2.05	0.040	R	21.94	0.003	68.0
bb vs. bB	Overall	966	856	8	0.96 (0.77-1.20)	0.33	0.740	R	5.01	0.660	0.0
bb + bB vs. BB	Overall	1210	1160	8	1.81 (1.03-3.17)	2.07	0.040	R	23.82	0.001	71.0
bb + bB vs. BB	Asian	738	640	3	1.21 (0.67-2.32)	0.69	0.490	R	3.10	0.210	36.0
bb + bB vs. BB	Caucasian	472	520	5	2.43 (1.02-5.80)	2.00	0.050	R	19.52	<0.001	80.0
bb + bB vs. BB	Hypercalciuria	123	340	3	2.43 (1.36-4.35)	2.99	0.003	R	1.99	0.370	0.0
bb + bB vs. BB	Adult	1146	1070	7	1.80 (0.96-3.38)	1.83	0.070	R	23.65	<0.001	75.0
bb + bB vs. BB	HWE	909	820	5	1.36 (0.87-2.13)	1.35	0.180	R	4.90	0.300	18.0
bb + bB vs. BB	High quality	794	709	3	1.15 (0.75-1.72)	0.66	0.510	R	1.01	0.600	0.0
*Fo*kI											
ff vs. FF	Overall	1144	1243	14	1.59 (1.08-2.35)	2.34	0.020	R	47.26	<0.001	72.0
fF vs. FF	Overall	1647	1809	14	1.38 (0.96-2.00)	1.72	0.090	R	64.13	<0.001	80.0
ff vs. fF	Overall	1741	1804	14	1.04 (0.74-1.45)	0.22	0.830	R	56.68	<0.001	77.0
ff + fF vs. FF	Overall	2266	2418	14	1.48 (1.03-2.12)	2.12	0.030	R	68.26	<0.001	81.0
ff + fF vs. FF	Asian	2005	2188	12	1.32 (0.94-1.86)	1.62	0.110	R	44.62	<0.001	75.0
ff + fF vs. FF	Hypercalciuria	324	560	5	1.15 (0.81-1.63)	0.79	0.430	R	1.42	0.840	0.0
ff + fF vs. FF	Adult	2024	1743	11	1.71 (1.14-2.56)	2.61	0.009	R	59.01	<0.001	83.0
ff + fF vs. FF	HWE	1390	1125	7	1.05 (0.83-1.33)	0.39	0.700	R	8.54	0.200	30.0
ff + fF vs. FF	High quality	1513	1348	7	1.34 (0.83-2.16)	1.19	0.230	R	53.59	<0.001	85.0
*TaqI*											
tt vs. TT	Overall	1144	1478	13	1.24 (0.92-1.68)	1.40	0.160	R	12.19	0.430	2.0
tT vs. TT	Overall	1558	1786	13	1.33 (1.04-1.70)	2.28	0.020	R	21.41	0.040	44.0
tt vs. Tt	Overall	806	624	13	0.92 (0.61-1.38)	0.40	0.690	R	21.56	0.040	44.0
tt + tT vs. TT	Overall	1744	1944	13	1.30 (1.08-1.55)	2.79	0.005	R	13.54	0.330	11.0
tt + tT vs. TT	Asian	1110	1386	6	1.39 (1.11-1.73)	2.87	0.004	R	4.32	0.500	0.0
tt + tT vs. TT	Caucasian	644	558	7	1.21 (0.84-1.72)	1.02	0.310	R	10.06	0.120	40.0
tt + tT vs. TT	Hypercalciuria	176	183	5	1.56 (1.06-2.31)	2.24	0.030	R	2.47	0.650	0.0
tt + tT vs. TT	Adult	1481	1244	9	1.34 (1.05-1.71)	2.35	0.020	R	12.71	0.120	37.0
tt + tT vs. TT	HWE	1098	964	7	1.36 (1.07-1.77)	2.51	0.010	R	7.44	0.280	19.0
tt + tT vs. TT	High quality	1392	1676	8	1.27 (1.05-1.54)	2.43	0.010	R	9.23	0.240	24.0
*Apa*I-*TaqI* haplotype											
At vs. aT	Overall	830	1207	4	1.35 (1.09-1.68)	2.71	0.007	R	2.91	0.410	0.0
At vs. aT	Asian	644	961	3	1.53 (1.17-2.00)	3.14	0.002	R	0.35	0.840	0.0
At vs. aT	Adult	752	806	3	1.36 (1.03-1.80)	2.16	0.030	R	2.90	0.230	31.0

vs., versus; HWE, Hardy-Weinberg equilibrium; R, random-effects model.

#### ***VDR BsmI polymorphism***

**Figure 4:**
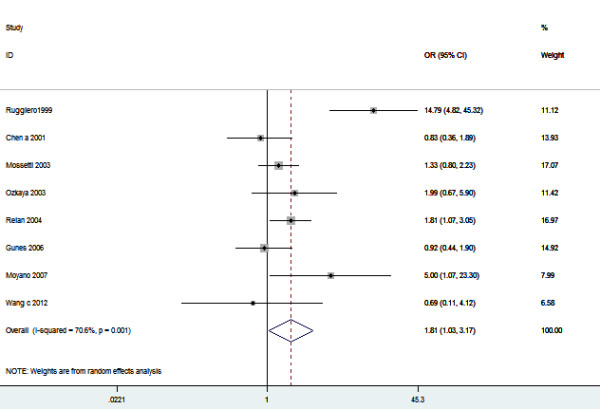
**Meta-analysis for the association between urolithiasis risk and the *VDR BsmI *polymorphism.**

**Figure 5:**
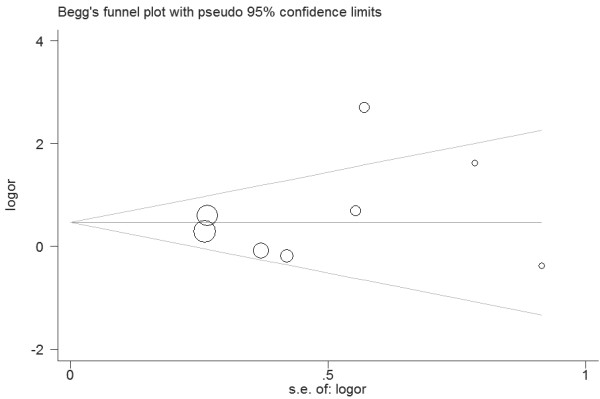
**Funnel plot for publication bias in selection of studies on the *VDR BsmI *polymorphism polymorphism.**

Eight studies (1210 cases and 1160 controls) that identified the association between *VDR BsmI* polymorphism and urolithiasis risk were included in this meta-analysis. The estimated OR1, OR2 and OR3 were 1.79, 1.85, and 0.96, respectively (Table 2). These estimates suggested a dominant genetic model. The pooled OR was 1.81 (95% CI 1.03 – 3.17, *P* = 0.04) (Figure 4). In the subgroup analysis by ethnicity, a marginally significant association was found among Caucasians (OR = 2.43, 95% CI 1.02 – 5.80, *P* = 0.05) but not among Asians (OR 1.21, 95% CI 0.67 – 2.32, *P* = 0.49) (Table 2). In addition, subgroup analysis in hypercalciuric patients showed significant increased risk of urolithiasis (OR 2.43, 95% CI 1.36 – 4.35, *P* = 0.003). In the stratified analysis by ethnicity, no significant association was found among adults (OR 0.80, 95% CI 0.96 – 3.38, *P* = 0.07). However, sensitivity analyses conducted by excluding the studies not in HWE or low quality studies did not find the significant association between *VDR BsmI* polymorphism and urolithiasis risk (Table 2). No publication bias was detected by funnel plot (Figure 5) and Egger’s test (*P* = 0.461).

#### ***VDR FokI polymorphism***

**Figure 6:**
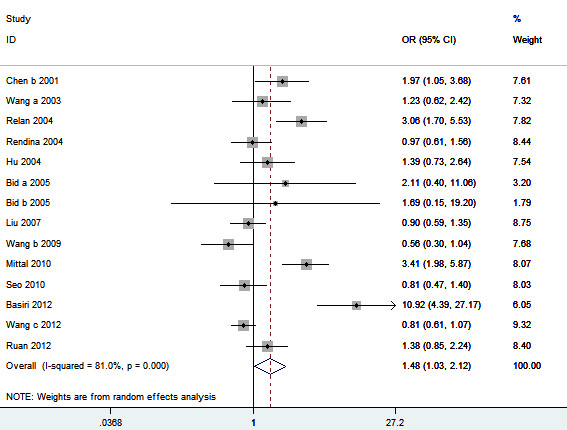
**Meta-analysis for the association between urolithiasis risk and the *VDR FokI *polymorphism.**

**Figure 7:**
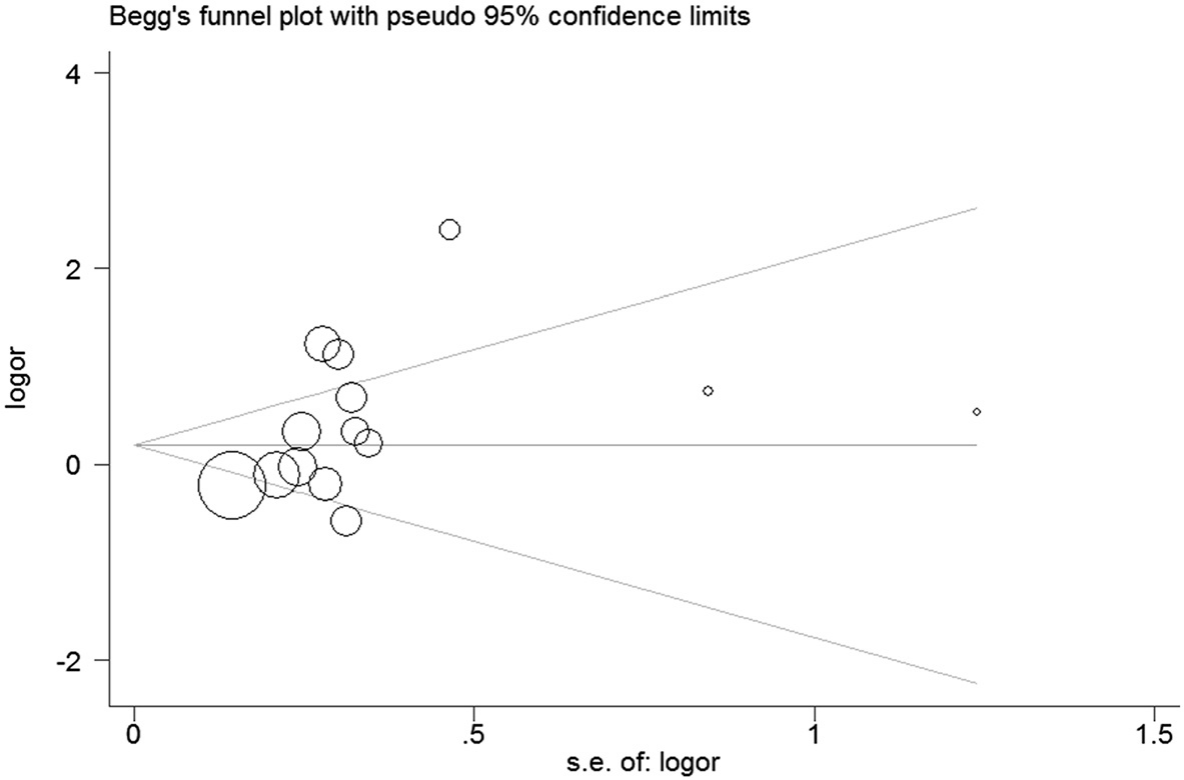
**Funnel plot for publication bias in selection of studies on the *VDR FokI *polymorphism.**

For the *VDR FokI* polymorphism, fourteen studies including 2266 cases and 2418 controls were included in this meta-analysis. OR1, OR2, and OR3 were 1.59, 1.38, and 1.04, respectively. These estimates suggested a dominant genetic model. Therefore, the original grouping was collapsed, and ff and fF were combined, in accordance with a dominant model, into a f carrier group, the latter of which was compared with the FF genotype group. The pooled OR was 1.48 (95% CI 1.03 – 2.12, *P* = 0.03) (Figure 6). In the subgroup analysis by ethnicity, no significant association was found among Asians (OR = 1.32, 95% CI 0.94 – 1.86, *P* = 0.11) (Table 2). In the subgroup analysis by the calciuria level, there was still no significant association between *VDR FokI* polymorphism and urolithiasis risk in patients with hypercalciuria (OR = 1.15, 95% CI 0.81 – 1.63, *P* = 0.43) (Table 2). Statistically significant increased urolithiasis risk was observed among adults group (OR = 1.71, 95% CI 1.14 – 2.56, *P* = 0.009). Sensitivity analyses found that the significant result was altered when the low quality studies or studies with Hardy-Weinberg disequilibrium were omitted (Table 2). The shape of the funnel plot seemed symmetrical (Figure 7). Egger’s test did not show evidence of publication bias (*P* = 0.081).

#### ***VDR TaqI polymorphism***

**Figure 8:**
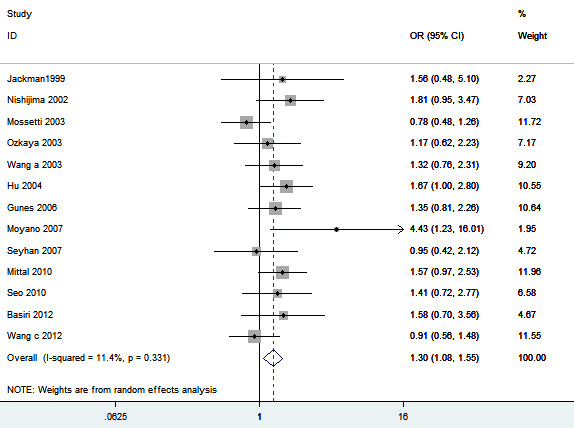
**Meta-analysis for the association between urolithiasis risk and the *VDR TaqI *polymorphism.**

**Figure 9:**
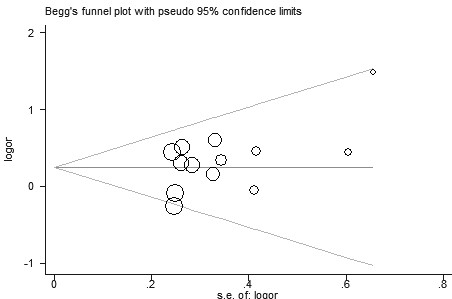
**Funnel plot for publication bias in selection of studies on the *VDR TaqI *polymorphism.**

Thirteen studies including 1744 patients and 1944 controls addressed the association between *VDR TaqI* polymorphism and urolithiasis. The estimated OR1, OR2 and OR3 were 1.24, 1.33, and 0.92, respectively. These pooling estimates suggested a dominant genetic model. The pooled OR in the dominant genetic model was 1.30 (95% CI 1.08 – 1.55, *P* = 0.005) (Figure 8). In the stratified analysis, statistically significant increased urolithiasis risk was observed among Asians (OR = 1.39, 95% CI 1.11 – 1.73, *P* = 0.004) but not Caucasians (OR = 1.21, 95% CI 0.84 – 1.72, *P* = 0.31). Furthermore, patients with hypercalciuria had a significant increase of the risk of developing urolithiasis (OR = 1.56, 95% CI 1.06 – 2.31, *P* = 0.03). In addition, significantly increased urolithiasis risks were found among adults (OR = 1.34, 95% CI 1.05 – 1.71, *P* = 0.02). Sensitivity analysis did not change the result (Table 2). The funnel plot appeared to be symmetrical (Figure 9). Egger’s test were performed to estimate the publication bias. Publication bias was not observed (*P* = 0.122).

### VDR ApaI-TaqI haplotype

**Figure 10:**
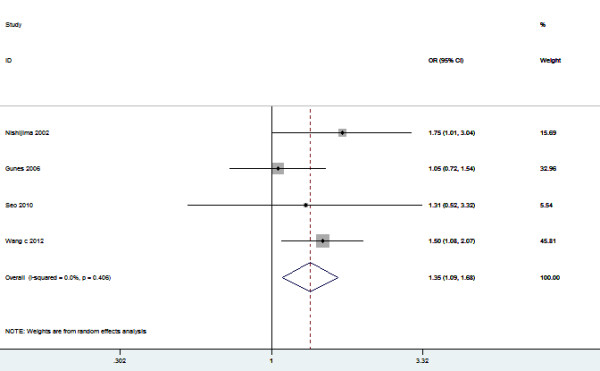
**Meta-analysis for the association between urolithiasis risk and the *VDR ApaI*-*TaqI *haplotype.**

**Figure 11:**
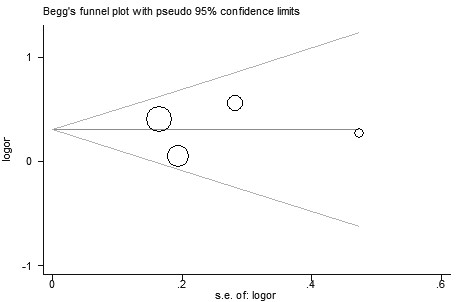
**Funnel plot for publication bias in selection of studies on the *VDR ApaI*-*TaqI *haplotype.**

In 4 out of 23 studies, the role of *VDR ApaI*-*TaqI* haplotype in urolithiasis risk was analyzed. The sample sizes in case group and control group were 830 and 1207, respectively. Carling et al. [37] reported that the At haplotype displayed higher levels of mRNA expression than aT haplotype. Thus, the effect of these two haplotypes on the risk of urolithiasis was evaluated in this meta-analysis. As shown in Figure 10, the pooling estimate showed a significant increased risk of developing urolithiasis in subjects with the At haplotype (OR = 1.35, 95% CI 1.09 – 1.68, *P* = 0.007). In the subgroup analysis by ethnicity, a significant association was found among Asians (OR = 1.53, 95% CI 1.17 – 2.00, *P* = 0.002). In the stratified analysis of age group, we also found a significant association (OR = 1.36, 95% CI 1.03 – 1.80, *P* = 0.03). Symmetrical funnel plot was obtained (Figure 11). Publication bias was not observed (*P* = 0.887).

## Discussion

This present meta-analysis systematic investigated the associations between *VDR* polymorphisms and urolithiasis risk. We found that *ApaI* polymorphism was a risk factor for developing urolithiasis. This result indicated that the carriers of the AA or Aa genotype had a 34% increased urolithiasis risk compared to those individuals with the aa genotype. Sensitivity analysis did not change this result, suggesting the solidity of our result. In the stratified analysis by ethnicity, the significant association was found in Asian population but not in Caucasian population. In addition, *BsmI* and *FokI* polymorphisms were also significantly associated with urolithiasis risk in the overall population. However, results from sensitivity analyses suggested that these results were not statistically robust. Therefore, these results should be interpreted with caution and more studies are needed to evaluate the effect of *BsmI* and *FokI* polymorphisms on urolithiasis risk. Specifically, we noted that hypercalciuric patients who harbored b allele may have increased urolithiasis risk. As for the *TaqI* polymorphism, a significant association was observed between this polymorphism and the risk of urolithiasis. Sensitivity analyses further strengthened the validity of this result. When subgroup analysis was conducted according to the ethnicity, the significant association was showed in Asians and lack of a significant association was detected in Caucasian population. In the hypercalciuria subgroup analysis, a 56% increased risk of urolithiasis was found in patients with hypercalciuria. Collectively, these results suggested that *VDR BsmI* and *TaqI* polymorphisms may play unique roles in the etiology of hypercalciuric urolithiasis. However, because the number of studies included in the hypercalciuria subgroup analyses was small, the results lacked sufficient reliability to confirm these associations in a definitive manner. Thus, future studies with larger sample sizes are needed to confirm our results. Furthermore, we assessed the association between the haplotypes of the *ApaI* and *TaqI* polymorphisms and urolithiasis risk. The result from this meta-analysis suggested that At haplotype may play a role in urolithiasis susceptibility, especially in Asians. Taken together, our results exhibited significant associations between *ApaI* and *TaqI* polymorphisms and urolithiasis risk in Asian population but not in Caucasian population. There are at least four reasons to explain the ethnic difference. First, the number of Caucasian patients was small. There were only 384 and 644 Caucasian patients for these two polymorphisms, respectively. It was therefore possible that the observed ethnic difference was due to chance. More studies with Caucasian population are required to validate the effect of ethnic differences. Second, higher heterogeneity was observed in the Caucasians subgroup (*I*^2^ = 40%) but not in the Asians subgroup (*I*^2^ = 0%) in *TaqI* polymorphism. This may distort the result. Third, urolithiasis is a complex disease. Both genetic and environmental factors could affect the risk of urolithiasis in different populations. It is possible that different urolithiasis risks in Asians and Caucasians were due to exposure to various environmental factors. To data, there was no reported study which was performed to assess the effect of *VDR-*environment interactions on urolithiasis risk in different ethnicities and regions. Therefore, epidemiologic studies should be designed to examine these associations in the future. Fourth, ethnic difference in the *VDR* gene allele frequencies may also result in this difference. For example, the A allele of the *ApaI*, occured with lower frequency in Caucasians when compared to Asians, while the t allele of the *TaqI* had a higher frequency in Asians compared to Caucasians [38]. We also performed subgroup analyses by age group. There were still significant associations in the adults group except in *BsmI* polymorphism. These results indicated that these polymorphisms were significantly associated with increased urolithiasis risk in adults. We did not evaluate the associations between *VDR* polymorphisms and urolithiasis risk in children due to insufficient data. More studies using children population are needed to determine the associations between *VDR* polymorphisms and urolithiasis risk.

A considerable weight of evidence supporting a role for VDR in urolithiasis was derived from GHS rats. Experimental studies using GHS rats showed increased levels of VDR in intestine, bones and kidneys [6,39]. Moreover, Karnauskas et al. [40] found that prolongation of VDR half-life increased VDR tissue levels and mediated VDR-regulated genes that led to hypercalciuria. Recently, using microRNA targeting *VDR*, Xi and colleagues silenced the *VDR* gene in the kidneys of GHS rats [41]. They demonstrated *VDR* knockdown in the kidney can upregulate the expression of transient receptor potential vanilloid receptor subtype 5 (TRPV5) in GHS rats [41]. Previous study found that mice lacking TRPV5 exhibited reduced calcium reabsorption, which caused severe hypercalciuria [42]. In addition, hypercalciuria increased the risk for calcium oxalate nephrolithiasis and occurred in up to 50% of nephrolithiasis patients [43,44]. Therefore, high VDR level might contribute to the development of urolithiasis. *VDR* was one of the most studied candidate genes for urolithiasis. Carling et al. [37] showed that the individuals exhibiting the BB, AA, or tt genotypes had significantly higher VDR levels than those with homozygous for the b, a, or T alleles. They also found those exhibiting the baT haplotype demonstrated a relative lower VDR mRNA level than subjects with non-baT haplotype [37]. Furthermore, Yamagata et al. [45] showed the VDR mRNA levels of allele t were significantly higher than those of allele T in peripheral blood mononuclear cell (PBMC). Thus, it is biologically plausible that subjects with the B, A, or t alleles may have increased risk of urolithiasis. Our findings supported this speculation and the At haplotype carriers had increased urolithiasis risk than the aT haplotype carriers. However, we found that b allele carriers, but not B allele carriers, were associated with urolithiasis, although this result was not robust. Notablely, hypercalciuric patients with b allele were seemed to have higher urolithiasis risk in the subgroup analysis. This result agreed with the report of Ferreira et al. [46], who also found that bb homozygous for VDR polymorphism was overrepresented in hypercalciuric stone formers. Additionally, Relan and co-authors reported that subjects with the bb genotype exhibited a higher urinary calcium excretion than the BB genotype [18]. Ruggiero et al. [10] found the similar phenomenon. The altered function of the *VDR* gene for the *BsmI* polymorphism may be associated with higher urinary calcium excretion. Hypercalciuria was a risk factor of urolithiasis. It was thus possible that b allele carriers may have higher urolithiasis risk than B allele carriers. As for the *FokI* polymorphism, Jurutka et al. [47] suggested that F variant possessed elevated transcriptional activity compared with the f variant. However, there was no difference between these two variants on the VDR expression [47]. Results from our study suggested that there was a significant association between f variant and urolithiasis. But the mechanism was unknown. The functional studies of *VDR FokI* polymorphism are still required. In addition, more studies are needed to confirm our results.

Our meta-analysis included a total of 23 case–control studies with 3046 cases and 3206 controls, while a previous meta-analysis by Lin et al. [48] only included 17 case–control studies with 2046 cases and 2303 controls. They found significant associations between *FokI* and *TaqI* polymorphisms and urolithiasis risk. However, we found *ApaI* and *TaqI* polymorphisms were significantly associated with urolithiasis risk. More case–control studies were included in our meta-analysis than Lin’s study. Therefore, our study may be more powerful and the conclusion might be more reliable. This reason might explain the difference between these two meta-analyses. In addition, our study had some advantages. First, Lin and coworkers tested multiple genetic models, including allelic comparison, a dominant model, and a recessive model. However, they did not correct for multiple comparisons or give biologic rationale for the choice of these genetic models. When the underlying genetic model is unknown, it is better to use pairwise comparisons of the three genotypes to find the best genetic model [34]. We used this method to avoide the problem of multiple comparisons. Second, it was the first time studying the *VDR*-hypercalciuria interactions on urolithiasis risk. Third, we explored the haplotype effect of *ApaI* and *TaqI* polymorphisms on the susceptibility to urolithiasis. Finally, the methodological issues for meta-analysis, such as, quality assessment was well investigated.

We should point out the importance of heterogeneity and publication bias, which might influence the results of meta-analysis. In our study, significant heterogeneity was found in the *Bsm*I and *FokI* polymorphisms. Subgroup analysis was used to explore the sources of heterogeneity. After subgroup analysis by the calciuria level, the heterogeneity was effectively disappeared in hypercalciuric patients. Thus, it could be presumed that the relatively large heterogeneity mainly resulted from the calciuria level. In addition, funnel plot and Egger’s tests were used to find potential publication bias. No significant publication bias was detected.

Some limitations of our study should be addressed. First, the number of studies that were included in this meta-analysis was moderate. It was possible that some relevant published studies or unpublished studies with negative results were missed. Second, all of the studies were performed in Asians and Caucasians; thus, our results may be applicable only to these ethnic groups. Third, this study did not address gene-gene and gene-environment interactions, because insufficient information could be extracted from the original publications. Fourth, the data of the stone types and biochemical profiles was not showed in our meta-analysis because of insufficient information from the primary publications. Finally, we could not investigate the association between the haplotypes of the *ApaI*, *BsmI*, and *TaqI* polymorphisms and urolithiasis risk.

## Conclusions

To our knowledge, this was the most comprehensive meta-analysis to assess the relationship between the *VDR* polymorphisms and urolithiasis susceptibility. Our results indicated that the *ApaI* and *TaqI* polymorphisms were associated with the risk of urolithiasis. Haplotype analysis suggested that *ApaI-TaqI* haplotypes conferred the susceptibility to urolithiasis. Future large-scale studies with more ethnic groups are needed to validate our findings. Gene-gene and gene-environment interactions should also be considered in future studies.

## Competing interests

The authors have declared that no competing interests exist.

## Authors’ contributions

PZ and WN collected the literature data, developed the statistical model, carried out the software implementation and drafted the manuscript. All authors read and approved the final manuscript.

## Pre-publication history

The pre-publication history for this paper can be accessed here:

http://www.biomedcentral.com/1471-2350/14/104/prepub

## References

[B1] Rivers K Shetty S Menon M When and how to evaluate a patient with nephrolithiasis Urol Clin North Am 2000 27 2 203 213 10.1016/S0094-0143(05)70251-2 10778464

[B2] Stamatelou KK Francis ME Jones CA Nyberg LM Curhan GC Time trends in reported prevalence of kidney stones in the United States: 1976–1994 Kidney Int 2003 63 5 1817 1823 10.1046/j.1523-1755.2003.00917.x 12675858

[B3] Goldfarb DS Fischer ME Keich Y Goldberg J A twin study of genetic and dietary influences on nephrolithiasis: a report from the Vietnam Era Twin (VET) registry Kidney Int 2005 67 3 1053 1061 10.1111/j.1523-1755.2005.00170.x 15698445

[B4] Curhan GC Willett WC Rimm EB Stampfer MJ Family history and risk of kidney stones J Am Soc Nephrol 1997 8 10 1568 1573 9335385 10.1681/ASN.V8101568

[B5] Elkoushy MA Sabbagh R Unikowsky B Andonian S Prevalence and metabolic abnormalities of vitamin D-inadequate patients presenting with urolithiasis to a tertiary stone clinic Urology 2012 79 4 781 785 10.1016/j.urology.2011.09.004 22035763

[B6] Yao J Kathpalia P Bushinsky DA Favus MJ Hyperresponsiveness of vitamin D receptor gene expression to 1, 25-dihydroxyvitamin D3: a new characteristic of genetic hypercalciuric stone-forming rats J Clin Invest 1998 101 10 2223 2232 10.1172/JCI1164 9593778 PMC508810

[B7] Favus MJ Karnauskas AJ Parks JH Coe FL Peripheral blood monocyte vitamin D receptor levels are elevated in patients with idiopathic hypercalciuria J Clin Endocrinol Metab 2004 89 10 4937 4943 10.1210/jc.2004-0412 15472188

[B8] Vezzoli G Terranegra A Arcidiacono T Soldati L Genetics and calcium nephrolithiasis Kidney Int 2010 80 6 587 593 20962745 10.1038/ki.2010.430

[B9] Morrison NA Yeoman R Kelly PJ Eisman JA Contribution of trans-acting factor alleles to normal physiological variability: vitamin D receptor gene polymorphism and circulating osteocalcin Proc Natl Acad Sci U S A 1992 89 15 6665 6669 10.1073/pnas.89.15.6665 1353882 PMC49563

[B10] Ruggiero M Pacini S Amato M Aterini S Chiarugi V Association between vitamin D receptor gene polymorphism and nephrolithiasis Miner Electrolyte Metab 1999 25 3 185 190 10.1159/000057443 10436404

[B11] Jackman SV Kibel AS Ovuworie CA Moore RG Kavoussi LR Jarrett TW Familial calcium stone disease: TaqI polymorphism and the vitamin D receptor J Endourol 1999 13 4 313 316 10.1089/end.1999.13.313 10405913

[B12] Chen WC Chen HY Hsu CD Wu JY Tsai FJ No association of vitamin D receptor gene BsmI polymorphisms with calcium oxalate stone formation Mol Urol 2001 5 1 7 10 10.1089/109153601750124203 11689145

[B13] Chen WC Chen HY Lu HF Hsu CD Tsai FJ Association of the vitamin D receptor gene start codon Fok I polymorphism with calcium oxalate stone disease BJU Int 2001 87 3 168 171 10.1046/j.1464-410x.2001.02074.x 11167636

[B14] Nishijima S Sugaya K Naito A Morozumi M Hatano T Ogawa Y Association of vitamin D receptor gene polymorphism with urolithiasis J Urol 2002 167 5 2188 2191 10.1016/S0022-5347(05)65126-9 11956476

[B15] Mossetti G Vuotto P Rendina D Numis FG Viceconti R Giordano F Cioffi M Scopacasa F Nunziata V Association between vitamin D receptor gene polymorphisms and tubular citrate handling in calcium nephrolithiasis J Intern Med 2003 253 2 194 200 10.1046/j.1365-2796.2003.01086.x 12542560

[B16] Ozkaya O Söylemezoğlu O Misirlioğlu M Gönen S Buyan N Hasanoğlu E Polymorphisms in the vitamin D receptor gene and the risk of calcium nephrolithiasis in children Eur Urol 2003 44 1 150 154 10.1016/S0302-2838(03)00206-9 12814692

[B17] Wang S Liu J Hu S Ye Z Association of vitamin D receptor gene polymorphisms with calcium oxalate calculus disease J Huazhong Univ Sci Technol 2003 23 1 38 41 10.1007/BF02829458

[B18] Relan V Khullar M Singh SK Sharma SK Association of vitamin d receptor genotypes with calcium excretion in nephrolithiatic subjects in northern India Urol Res 2004 32 3 236 240 15205858 10.1007/s00240-004-0414-x

[B19] Rendina D Mossetti G Viceconti R Sorrentino M Castaldo R Manno G Guadagno V Strazzullo P Nunziata V Association between vitamin D receptor gene polymorphisms and fasting idiopathic hypercalciuria in recurrent stone-forming patients Urology 2004 64 4 833 838 10.1016/j.urology.2004.05.013 15491743

[B20] Hu SQ Liu JH Wang SG Cao ZG Wu W Relationship between vitamin D receptor allele polymorphism and calcium oxalate stone disease Chin J Urol 2004 25 3 155 158

[B21] Bid HK Chaudhary H Mittal RD Association of vitamin-D and calcitonin receptor gene polymorphism in paediatric nephrolithiasis Pediatr Nephrol 2005 20 6 773 776 10.1007/s00467-005-1846-4 15856322

[B22] Bid HK Kumar A Kapoor R Mittal RD Association of vitamin D receptor-gene (FokI) polymorphism with calcium oxalate nephrolithiasis J Endourol 2005 19 1 111 115 10.1089/end.2005.19.111 15735395

[B23] Gunes S Bilen CY Kara N Asci R Bagci H Yilmaz AF Vitamin D receptor gene polymorphisms in patients with urolithiasis Urol Res 2006 34 1 47 52 10.1007/s00240-005-0033-1 16397775

[B24] Liu CC Huang CH Wu WJ Huang SP Chou YH Li CC Chai CY Wu MT Association of vitamin D receptor (Fok-I) polymorphism with the clinical presentation of calcium urolithiasis BJU Int 2007 99 6 1534 1538 10.1111/j.1464-410X.2007.06792.x 17419705

[B25] Moyano MJ de Tejada MJ G García Lozano R Moruno R Ortega R Martí V Sánchez Palencia R Miranda MJ Palma A Pérez Cano R Alterations in bone mineral metabolism in patients with calcium kidney stone disease and polymorphism of vitamin D receptor. Preliminary results Nefrologia 2007 27 6 694 703 18336098

[B26] Seyhan S Yavascaoglu I Kilicarslan H Dogan HS Kordan Y Association of vitamin D receptor gene Taq I polymorphism with recurrent urolithiasis in children Int J Urol 2007 14 12 1060 1062 10.1111/j.1442-2042.2007.01899.x 18036039

[B27] Wang QZ Qian B Ding GF Zheng LY Vitamin D receptor gene polymorphisms in Chinese uygur patients with uroHthiasis in south Xinjian J Pract Med 2009 25 17 2805 2807

[B28] Mittal RD Mishra DK Srivastava P Manchanda P Bid HK Kapoor R Polymorphisms in the vitamin D receptor and the androgen receptor gene associated with the risk of urolithiasis Indian J Clin Biochem 2010 25 2 119 126 10.1007/s12291-010-0023-0 23105897 PMC3453098

[B29] Seo IY Kang IH Chae SC Park SC Lee YJ Yang YS Ryu SB Rim JS Vitamin D receptor gene Alw I, Fok I, Apa I, and Taq I polymorphisms in patients with urinary stone Urology 2010 75 4 923 927 10.1016/j.urology.2009.10.006 20018354

[B30] Basiri A Shakhssalim N Houshmand M Kashi AH Azadvari M Golestan B Mohammadi Pargoo E Pakmanesh H Coding region analysis of vitamin D receptor gene and its association with active calcium stone disease Urol Res 2012 40 1 35 40 10.1007/s00240-011-0399-1 21814771

[B31] Wang S Wang X Wu J Lin Y Chen H Zheng X Zhou C Xie L Association of vitamin D receptor gene polymorphism and calcium urolithiasis in the Chinese Han population Urol Res 2012 40 4 277 284 10.1007/s00240-011-0438-y 22116536

[B32] Ruan L Li ZM Zheng RG Huang WS Shi GQ Li G Li S Luo B Relationship between vitamin D receptor *Fo*kI polymorphism and calcium oxalate stone disease in Guangzhou Chinese patients Guangdong Med J 2012 33 1 84 85

[B33] Clark MF Baudouin SV A systematic review of the quality of genetic association studies in human sepsis Intensive Care Med 2006 32 11 1706 1712 10.1007/s00134-006-0327-y 16957907

[B34] Thakkinstian A McElduff P D’Este C Duffy D Attia J A method for meta-analysis of molecular association studies Statist Med 2004 24 9 1291 1306 10.1002/sim.2010 15568190

[B35] Nie W Chen J Xiu Q Cytotoxic T-lymphocyte associated antigen 4 polymorphisms and asthma risk: a meta-analysis PloS ONE 2012 7 7 e42062 10.1371/journal.pone.0042062 22844542 PMC3406027

[B36] Egger M Smith GD Schneider M Minder C Bias in meta-analysis detected by a simple, graphical test BMJ 1997 315 7109 629 634 10.1136/bmj.315.7109.629 9310563 PMC2127453

[B37] Carling T Rastad J Åkerström G Westin G Vitamin D receptor (VDR) and parathyroid hormone messenger ribonucleic acid levels correspond to polymorphic VDR alleles in human parathyroid tumors J Clin Endocrinol Metab 1998 83 7 2255 2259 10.1210/jc.83.7.2255 9661591

[B38] Uitterlinden AG Fang Y van Meurs JBJ Pols HAP van Leeuwen JPTM Genetics and biology of vitamin D receptor polymorphisms Gene 2004 338 2 143 156 10.1016/j.gene.2004.05.014 15315818

[B39] Bai S Wang H Shen J Zhou R Bushinsky DA Favus MJ Elevated vitamin D receptor levels in genetic hypercalciuric stone-forming rats are associated with downregulation of Snail J Bone Miner Res 2010 25 4 830 840 19929616 10.1359/jbmr.091010 PMC3153334

[B40] Karnauskas AJ van Leeuwen JP van den Bemd GJ Kathpalia PP DeLuca HF Bushinsky DA Favus MJ Mechanism and function of high vitamin D receptor levels in genetic hypercalciuric stone‒forming rats J Bone Miner Res 2005 20 3 447 454 15746989 10.1359/JBMR.041120

[B41] Xi QL Wang SG Ye ZQ Zhu ZW Li C Bai J Yu X Liu JH Effect of silencing VDR gene in kidney on renal epithelial calcium transporter proteins and urinary calcium excretion in genetic hypercalciuric stone-forming rats Urology 2011 78 6 1442.e1441 1442.e1447 22137721 10.1016/j.urology.2011.08.051

[B42] Hoenderop JG van Leeuwen JP van der Eerden BC Kersten FF van der Kemp AW Mérillat AM Waarsing JH Rossier BC Vallon V Hummler E Bindels RJ Renal Ca2+ wasting, hyperabsorption, and reduced bone thickness in mice lacking TRPV5 J Clin Invest 2002 112 12 1906 1914 14679186 10.1172/JCI19826 PMC297001

[B43] Moe OW Kidney stones: pathophysiology and medical management Lancet 2006 367 9507 333 344 10.1016/S0140-6736(06)68071-9 16443041

[B44] Coe FL Evan A Worcester E Kidney stone disease J Clin Invest 2005 115 10 2598 2608 10.1172/JCI26662 16200192 PMC1236703

[B45] Yamagata M Nakajima S Tokita A Sakai N Yanagihara I Yabuta K Ozono K Analysis of the stable levels of messenger RNA derived from different polymorphic alleles in the vitamin D receptor gene J Bone Miner Res 1999 17 3 164 170 10.1007/s007740050080 10757675

[B46] Ferreira LG Pereira AC Heilberg IP Vitamin D receptor and calcium-sensing receptor gene polymorphisms in hypercalciuric stone-forming patients Nephron Clin Pract 2010 114 2 135 144 10.1159/000254386 19887834

[B47] Jurutka PW Remus LS Whitfield GK Thompson PD Hsieh JC Zitzer H Tavakkoli P Galligan MA Dang HT Haussler CA Haussler MR The polymorphic N terminus in human vitamin D receptor isoforms influences transcriptional activity by modulating interaction with transcription factor IIB Mol Endocrinol 2000 14 3 401 420 10.1210/me.14.3.401 10707958

[B48] Lin Y Mao Q Zheng X Chen H Yang K Xie L Vitamin D receptor genetic polymorphisms and the risk of urolithiasis: a meta-analysis Urol Int 2011 86 3 249 255 10.1159/000323949 21325790

